# Upregulation of Irisin and Vitamin D-Binding Protein Concentrations by Increasing Maternal 25-Hydrovitamin D Concentrations in Combination with Specific Genotypes of Vitamin D-Binding Protein Polymorphisms

**DOI:** 10.3390/nu14010090

**Published:** 2021-12-26

**Authors:** Spyridon N. Karras, Erdinç Dursun, Merve Alaylıoglu, Duygu Gezen-Ak, Fatme Al Anouti, Stefan Pilz, Pawel Pludowski, Edward Jude, Kalliopi Kotsa

**Affiliations:** 1 Division of Endocrinology and Metabolism and Diabetes Center, 1st Department of Internal Medicine, Medical School, Aristotle University of Thessaloniki, AHEPA University Hospital, 55535 Thessaloniki, Greece; kalmanthou@yahoo.gr; 2 Brain and Neurodegenerative Disorders Research Laboratories, Department of Medical Biology, Cerrahpasa Faculty of Medicine, Istanbul University-Cerrahpasa, Istanbul 34381, Turkey; erdincdu@gmail.com (E.D.); merve.alaylioglu@hotmail.com (M.A.); duygugezenak@gmail.com (D.G.-A.); 3 Department of Neuroscience, Institute of Neurological Sciences, Istanbul University-Cerrahpasa, Istanbul 34381, Turkey; 4 Department of Health Sciences, College of Natural and Health Sciences, Zayed University, Abu Dhabi 144534, United Arab Emirates; Fatme.AlAnouti@zu.ac.ae; 5 Division of Endocrinology and Diabetology, Department of Internal Medicine, Medical University of Graz, A-8036 Graz, Austria; stefan.pilz@medunigraz.at; 6 Department of Biochemistry, Radioimmunology and Experimental Medicine, The Children’s Memorial Health Institute, 04730 Warsaw, Poland; pludowski@yahoo.com; 7 Department of Endocrinology, Tameside Hospital NHS Foundation Trust, Ashton-under-Lyne OL6 9RW, UK; ejude99@yahoo.co.uk

**Keywords:** vitamin D, pregnancy, neonatal health, functional polymorphism

## Abstract

Dyshomeostasis of vitamin D-binding protein (VDBP) has been implicated in the pathogenesis of various pregnancy complications, including preeclampsia, preterm birth, gestational diabetes, and adverse metabolic profiles in the offspring. VDBP polymorphisms have been consistently reported to contribute to this intriguing interplay. Until recently, the effects of VDBP polymorphism heterogeneity on maternal and neonatal adipomyokine profiles have not been investigated, specifically after incorporating the different maternal and neonatal 25-hydroxyvitamin D concentration cut-offs at birth. We aimed to investigate the potential effects of maternal and neonatal VDBP polymorphisms on adiponectin, irisin, and VDBP concentrations at birth, according to different cut-offs of vitamin D status, in maternal–neonatal dyads recruited from the sunny region of Northern Greece. We obtained blood samples from 66 mother–child pairs at birth. Results indicated that (i) Neonatal serum biomarkers were not affected by any included neonatal VDBP polymorphism according to different cut-offs of neonatal vitamin D status at birth, (ii) neonatal VDBP concentration was elevated in neonates with maternal rs7041 GG genotype, (iii) maternal 25(OH)D at ≤75 nmol/L resulted in increased concentrations of maternal VBDP and irisin concentrations in women with CC genotype for rs2298850 and rs4588,whereas this effect was also evident for this cut-off for neonatal VDBP concentrations at birth for GC genotype for rs 7041, and (iv) no significant effect of neonatal VDBP polymorphisms was observed on neonatal VDBP, adiponectin, or irisin levels when stratified according to maternal 25(OH)D cut-offs. In conclusion, these findings confirm that among women with the combination of CC genotype for rs2298850 and rs4588, a specific high cut-off of maternal 25(OH)D results in increasing maternal VBDP concentrations, hence providing a mechanistic rationale for aiming for specific cut-offs of vitamin D after supplementation during pregnancy, in daily clinical practice.

## 1. Introduction 

Vitamin D-binding protein (VDBP) is considered as a crucial factor for vitamin D homeostasis [1,2] since it comprises the major biological parameter regulating the half-life of vitamin D in the systemic circulation. These effects are mediated by both serum VDBP concentrations and VDBP genotype, which affect bioavailable 25-hydroxyvitamin D[25(OH)D] [3,4]. VDBP has been also considered as a potent immuno-regulator implicated in the pathogenesis of autoimmune diseases, either through its effects on vitamin D equilibrium or via direct effects, which are mediated by VDBP concentrations [5]. 

VDBP dyshomeostasis has been linked to a plethora of complications, including gestational diabetes, preeclampsia, preterm birth, and adverse metabolic profiles in the offspring [6,7,8]. Our group has previously reported a strong association for VDBP concentrations with adipomyokines, adiponectin, and irisin, which are involved in energy regulation in both mothers and neonates [9]. We also confirmed this association between VDBP and adiponectin in healthy nonpregnant women, with no such association observed among men [10]. Vitamin D receptor and VDBP polymorphisms have been consistently reported to contribute to this intriguing interplay [11,12,13,14]. We have recently described that although vitamin D concentrations in the examined neonates were not impacted by maternal VDBP polymorphisms at birth, mothers with CC genotype for rs2298850 and CC genotype for rs4588 manifested higher 25(OH)D concentrations [11]. These effects were evident after adopting conventional international maternal or neonatal cut-offs for 25(OH)D concentrations. VDBP could be also considered as a molecule with significant metabolic functions regulating energy homeostasis. However, the potential effects of VDBP polymorphism heterogeneity on maternal and neonatal adipomyokine profiles remain largely unexplained, specifically after incorporating the effects of maternal and neonatal vitamin D status at birth.

Plasma half-life of 25(OH)D is approximately 3 weeks, according to previous reports [15]. It is a useful biomarker of environmental and physiological determinants of vitamin D status, including dietary and cutaneous synthesis, and is determined by CYP27B1 and CYP24A1 enzyme activity and all factors that influence the delivery and transport of 25(OH)D, including VDBP concentrations and genotypes [15].

Despite the sunny weather in Greece and the other Mediterranean countries, vitamin D deficiency is a major public health burden [16]. Moreover, a systematic review regarding hypovitaminosis D in the Mediterranean region including 2649 pregnant women revealed a prevalence range between 22.7% and 90.3% for vitamin D deficiency [17].We aimed to examine the potential effects of maternal and neonatal VDBP concentrations and polymorphisms on the specific adipomyokines adiponectin and irisin at birth, according to different cut-offs of vitamin D status, in maternal–neonatal dyads from the sunny region of Northern Greece.

## 2. Methods 

### 2.1. Inclusion and Exclusion Criteria 

A cohort of mother–child pairs at birth was included in the study. A detailed description of the enrollment has been previously described [18]. Informed consent was obtained from all mothers. The study was conducted from January 2018 to September 2019 and was granted ethical approval by the Bioethics Committee of the Aristotle University of Thessaloniki, Greece (approval number 1/19-12-2011). 

### 2.2. Demographic and Dietary Data—Biochemical and Hormonal Assays 

At enrollment, demographic and social characteristics were recorded. All dietary and demographic data of the cohort and methods of sampling have been reported previously [19]. Concentrations of 25(OH)D2 and 25(OH)D3 were determined using liquid chromatography–tandem mass spectrometry (LC–MS/MS), with lower limits of quantification (LLOQ); 25(OH)D2 (0.5 ng/mL), 25(OH)D3 (0.5 ng/mL), and the combination of the two vitamin D forms, as total 25(OH)D, were provided [20]. VDBP, irisin, and adiponectin were measured with enzyme-linked immunosorbent assay on a Synergy H1 Hybrid reader and Gen5 software (BioTek, Winooski, VT, USA): GC-Globulin/VDBP (AssayPro, St. Charles, MO, USA); irisin (My BioSource, San Diego, CA, USA); adiponectin (R&D Systems, Minneapolis, MN, USA). Intra-assay and inter-assay variance was <8% and <10% for adiponectin and <8% and <10% for irisin, respectively. Detection limits for assays were as follows: 0.098 μg/mL for VDBP, 3.12 ng/mL for irisin, and 0.039 μg/mL for adiponectin. We classified maternal and neonatal vitamin D status at birth according to the following: 25(OH)D ≤ 25 nmol/L (deficiency), 25–50 nmol/L (insufficiency), and 25(OH)D ≥ 50–75 nmol/L (sufficiency) [21,22].

### 2.3. VDBP Analysis 

DNA isolation was performed by QIAamp DNA Blood Mini Kit (Cat. No. 51304, QIAGEN, Hilden, Germany) according to the manufacturer’s protocol. Genotypes of VDBP rs2298850, rs4588, and rs7041 SNPs were determined by LightSNiP assay using simple probes (LightSNiP, TibMolBiol, Berlin, Germany) and LightCycler Fast Start DNA Master HybProbe Kit (Cat. No.12239272001, Roche Diagnostics, Mannheim, Germany). Real-time PCR (RT-PCR) was performed with LightCycler 480 Instrument II (Roche Diagnostics, Mannheim, Germany). Genotyping was performed as previously explained [23]. 

### 2.4. UVB Measurements

UVB data for the broad geographical region of Thessaloniki, Greece, were collected as described previously [18]. Mean UVB exposure during the previous 21 days (daily integral) before blood sample collection (estimated mean half-life of vitamin D) was calculated. 

### 2.5. Statistical Analysis 

All analyses that involved the distributions of genotypes of VDBP polymorphisms were analyzed with the chi-square (χ2) test, df:2 for genotypes. Significance was also confirmed with Cramer’s V/Kendall’s tau-c. One-way ANOVA and multiple-comparison tests were run to compare between mean values of the groups, either Tukey HSD or Dunett C, depending on the normality of the data set. Homogeneity of variances was checked with Levene’s Test for homogeneity of variances. Data and *p*-values were adjusted as needed by one-way analysis of covariance (ANCOVA) for maternal height (cm), BMI prepregnancy (kg/m^2^), BMI terminal (kg/m^2^), UVB, and weeks of gestation. All data are presented as mean ± SD. A *p*-value <0.05 was considered statistically significant. SPSS version 24.0” software (SPSS, Chicago, IL, USA) was used to perform statistical analysis.

## 3. Results 

### 3.1. Demographics of the Study Participants

The demographic characteristics of mothers–neonates cohort recruited in the study are shown in Table 1. There were 66 mothers with 28 female and 38 male neonates in total. Mean prepregnancy BMI (kg/m^2^) was 24.91 ± 4.81; while term pregnancy BMI (kg/m^2^) was 29.62 ± 5.80. Daily dietary vitamin D intake during the third trimester was 2.9 ± 1.2 mcg.

### 3.2. Distribution of Neonatal Adiponectin, Irisin, and VDBP Concentrations According to Maternal Vitamin D Status or Maternal VDBP Polymorphisms

Distributions of vitamin D status of neonatal adiponectin, irisin, and VDBP concentrations according to maternal vitamin D status are presented in Table 2. Data and *p*-values were adjusted for maternal height (cm), BMI prepregnancy (kg/m^2^), BMI at term (terminal has a different connotation) (kg/m^2^), UVB exposure, and weeks of gestation. The mean pre-conception BMI was 22.2 ± 3.3 kg/m^2^ (range 16.1–31.6), adjusted BMI was 22.4 ± 4.3 kg/m^2^ (range 13.5–35.5), and duration gestation was 37 to 42 weeks. The frequency distribution for the lower tertile of vitamin D status revealed that 47 mothers–neonates dyads had serum 25(OH)D ≥25 nmol/L, with concentrations of 8.97 ± 13.24 µg/mL and 7.28 ± 7.73 µg/mL for adiponectin, while 16 dyads had serum 25(OH)D ≤25 nmol/L, with concentrations of 20.56 ± 20.53 µg/mL and 20.95 ± 21.53 µg/mL for adiponectin. Differences across neonatal adiponectin concentrations according to maternal vitamin D status were remarkably significant (*p* = 0.048; adjusted = 0.003) with maternal 25(OH)D ≥25 nmol/L versus ≤25 nmol/L in the lower tertile, while there were no significant differences for the other biomarkers, irisin and VDBP, across the middle and upper tertiles. On the other hand, neonatal VDBP concentration in maternal rs7041 GG genotype was significantly increased compared to GT + TT genotype (GG genotype, *n*: 17, adjusted mean ± SD: 495.74 ± 279.57; GT + TT genotype, *n*: 47, adjusted mean ± SD: 295.44 ± 156.61; adjusted *p*: 0.02, power: 67%).

### 3.3. Neonatal Serum Biomarkers according to Neonatal Vitamin D Status at Birth and Neonatal VDBP Polymorphism

Neonatal serum adiponectin, irisin, and VDBP concentrations according to different neonatal vitamin D cut-offs at birth and neonatal VDBP polymorphisms are presented in Table 3. No significant effect was revealed for neonatal biomarkers, according to neonatal vitamin D status at birth and different SNPs and genotypes. The only remarkable effect was evident in neonates with 25(OH)D ≤ 75 nmol/L, which demonstrated higher irisin concentrations (233.56 ± 191.3 ng/mL),when harboring rs2298850 (CG + GG) (*p* = 0.04). However, results were nonsignificant after adjusting for maternal height (cm), BMI prepregnancy (kg/m^2^), BMI at term (kg/m^2^), UVB exposure, and weeks of gestation (*p* = 0.091).

### 3.4. Maternal Serum Biomarkers according to Maternal Vitamin D Status and Maternal VDBP Polymorphisms

Maternal serum adiponectin, irisin, and VDBP concentrations according to different maternal vitamin D cut-offs during delivery along with maternal VDBP polymorphisms are presented in Table 4. Mothers in the upper tertile of 25(OH)D (≤75 nmol/L),with rs2298850 (CC genotype),manifested higher VDBP concentrations (403.06 ± 64.72 µg/mL, *p* = 0.007) after multiple adjustments for maternal height (cm), BMI prepregnancy (kg/m^2^), BMI at term (kg/m^2^), UVB exposure, and weeks of gestation, compared with women in the middle and lower tertiles of 25(OH)D concentrations. Similar results were obtained for mothers with rs4588 (CC genotype) regarding VDBP concentrations (403.06 ± 64.72 µg/mL, *p* = 0.07), whereas the same SNP-genotype pattern revealed a similar effect on maternal irisin concentrations (508.57 ± 559.87 ng/mL, *p* = 0.03). 

### 3.5. Neonatal Serum Biomarkers According to Maternal Vitamin D Status and Maternal VDBP Polymorphisms

Neonatal serum adiponectin, irisin, and VDBP concentrations in the context of maternal VDBP polymorphisms and different maternal vitamin D cut-offs at birth are presented in Table 5. We observed that women with rs 7041 (genotype GC) delivered neonates with higher VDBP concentrations, at maternal concentrations of ≤50 nmol/L and ≤75 nmol/L (524.75 ± 331.56 µg/mL, *p* = 0.05 and 526.26 ± 282.39 µg/mL, *p* = 0.01, respectively). Significance was more pronounced (*p* = 0.01) with increasing maternal vitamin D concentrations, including a cut-off of 75 nmol/L.

### 3.6. Neonatal Serum Biomarkers According to Neonatal Vitamin D Status at Birth and Maternal VDBP Polymorphisms

Neonatal serum adiponectin, irisin, and VDBP concentrations according to different neonatal vitamin D cut-offs at birth and maternal VDBP polymorphisms are presented in Table 6. There were no significant effects of different maternal cut-offs of 25(OH)D according to neonatal vitamin D status and genetic profiles.

## 4. Discussion

We aimed to investigate interactions between VDBP polymorphisms and adiponectin (adipokine),irisin (myokine), and VDBP concentrations, according to different maternal and neonatal 25(OH)D cut-offs, in mother–neonate pairs at birth. To our knowledge, this is the first study on the effects of different VDBP polymorphisms on the adipo-myokine offspring profiles, in the sunny Mediterranean region of Northern Greece. Our results revealed the following:

(i) Neonatal serum biomarkers were not affected by any included neonatal VDBP polymorphism according to different cut-offs of neonatal vitamin D status at birth;
(ii) Neonatal VDBP concentration was increased in neonates with maternal rs7041 GG genotype;
(iii) Elevated maternal 25(OH)D at ≤75 nmol/L resulted in increased concentrations of maternal VBDP and irisin concentrations in women with CC genotype for rs2298850 and rs4588,whereas this effect was also evident for this cut-off for neonatal VDBP concentrations at birth for GC genotype for rs 7041;
(iv) No significant effect of neonatal VDBP polymorphisms was observed on neonatal VDBP, adiponectin, or irisin levels when stratified according to maternal 25(OH)D cut-offs.

We identified a specific type of functional polymorphism, in relation to vitamin D status, VDBP polymorphisms, and metabolic profiles of future mothers and their neonates. Higher maternal vitamin D status at birth affected concentrations of VDBP and irisin in women and neonates with a specific VDBP SNP-genotype pattern, indicating an intriguing interaction of a modifiable factor (maternal vitamin D status at birth) with a specific genetic background (SNPs for VDBP), resulting in differences in concentrations of metabolites, involved in energy regulation and immune response, such as those described for VDBP and irisin previously [9,10,11,12]. Apart from its functions as the primary carrier molecule of vitamin D, VDBP is considered as a critical regulator of the half-life of circulating vitamin D metabolites [1,2,3,4]. It is also considered as a potent immunomodulator during pregnancy, at placental and systemic level, inducing systemic and local maternal tolerance to paternal and fetal allo-antigens [7]. VDBP has been implicated in regulating gene expression of certain placental amino-transporters, which might be involved during in utero development in the control of amino acid transfer to the offspring [8]. The exact pathways of the association of VDBP dyshomeostasis and adverse pregnancy and offspring outcomes are still a matter of debate. In this regard, specific VDBP polymorphisms have been also consistently reported to contribute to this intriguing interplay of VDBP biodynamics and pregnancy complications [7,24].

A study among Chinese women showed that the risk allele-A of rs3733359 of VDBP gene was associated with a modest increase of risk for gestational diabetes mellitus (GDM) in the obese subgroup, where other SNPs demonstrated correlations with insulin and glucose homeostasis [25]. Another Chinese group reported that *GC* rs16847024 C > T was significantly associated with an increased risk of GDM, however 25(OH)D concentrations were not evaluated in most women included in the study; nevertheless, genes encoding VDBP were found to be associated with vitamin D status [26]. These interactions, however, should be carefully deciphered, since there are important ethnic variations, which do not necessarily confirm the above findings in other pregnant cohorts. In this regard, a prospective large case-control study from Norway evaluated potential interactions of VDBP and its polymorphisms in pregnant women at 18th week of gestation and after delivery, withT1D risk of offspring [27]. Although higher VDBP concentrations at term were associated with lower risk of T1D in the offspring, no effects of VDBP polymorphisms were evident. Absence or attenuation of the prominent physiological increase of VDBP concentrations during pregnancy has been also reported in women whose offspring later developed T1D from the same region [28]. A recent study from Sweden also investigated associations of gene polymorphisms of vitamin D metabolism with markers of insulin resistance and secretion with regard to the development of GDM. No associations of SNPs for VDBP and postpartum diabetes in women with a history of GDM, after multiple adjustments, were found [29]. It becomes clear that available findings on the field are largely affected by ethnic heterogeneity, highlighting the importance of national or regional data from homogeneous populations.

Similar findings were reported for rs4588 (CC genotype) and rs 7041 (TT genotype) in women with preeclampsia, compared to normotensive pregnant women from South Africa [30]. Results from United States reported that a variant in the *GC* flanking region (rs13150174) and a *GC* missense mutation in rs7041 were correlated with differences in log-transformed 25(OH)D concentration. A meta-analysis conducted by the same authors, also revealed that the minor allele for rs7041 was associated with elevated 25(OH)D concentrations and rs4588 was correlated with reduced 25(OH)D concentrations, among pregnant women [31]. The A-allele of the rs7041 polymorphism of the VDBP gene was also associated with a reduction in circulating 25(OH)D3 (difference in nmol/L) per allele of −5.48, and similar findings were observed for the T-allele of the rs4588 polymorphism (difference in nmol/L) per allele of −6.32 in a pregnant cohort from Northern Europe [28].

In addition, Chinese pregnant women with VDBP Gc-1f and Gc-1s genotypes had elevated plasma 25(OH)D concentrations compared to women with Gc-2 genotypes [32]. Similar results were obtained by a large cohort of 2658 women from the Zhoushan Pregnant Women Cohort study in China. Mutations of rs2298849 and rs7041 on the GC gene were respectively associated with higher 25(OH)D in the first and third trimesters, whereas mutations of seven SNPs (rs1155563, rs16846876, rs17467825, rs2282679, rs2298850, rs3755967, and rs4588) on the GC gene were respectively associated with lower 25(OH)D both in the first and in the third trimester. These effects were modified by season and vitamin D supplementation [33], which was an exclusion condition in our study. Vitamin D-binding protein polymorphisms have been shown to regulate attained 25(OH)D concentrations after the use of supplements during pregnancy. In this regard, a positive association between GC rs2282679 polymorphism and the achieved 25(OH)D status was noted following gestational cholecalciferol supplementation [34]. Recently, a multi-ethnic Asian genome-wide association study analysis pertaining to a birth cohort of three ethnicities identified rs4588 and its defining haplotype as a risk factor for low antenatal and cord blood vitamin D [35]. We have also recently reported that mothers with a CC genotype for rs2298850 and a CC genotype for rs4588 demonstrated higher 25(OH)D concentrations during delivery, confirming these findings in a Southern European pregnant population [11].

Irisin has been involved in the regulation of glucose homeostasis during pregnancy and neonatal body composition [36,37,38]. Irisin concentrations demonstrated a relationship with anthropometric measurements inappropriate for gestational-age infants, whereas low irisin concentrations in maternal serum were reported in pregnancies that developed preeclampsia and isolated intrauterine growth retardation [37,38,39,40]. We have previously described that maternal VDBP concentrations demonstrate a strong positive correlation with maternal adiponectin and irisin concentrations, after adjustments for weeks of gestation, maternal age, and BMI [9,10]. Further investigations are required to decipher the exact dynamic pathways of VDBP, adiponectin, and irisin during pregnancy and their effects on pregnancy complications and offspring body anthropometry.

During pregnancy, the binding affinity of VDBP for vitamin D metabolites is reduced to compensate for the maternal and neonatal higher demand for calcium and elevated VDBP concentrations, with almost two-fold increases between the second and third trimesters during fetal development [6]. Moreover, physiological hemodilution might affect maternal serum 25(OH)D levels due to maternal plasma volume expansion. Inflammation, placental functions, and iron and calcium metabolism also contribute to the peculiarities of vitamin D metabolism during pregnancy. In this regard, free 25(OH)D may be a better indicator compared to total 25(OH)D since it remains comparable to levels reported in nonpregnant women [41]. The analytical significance of several vitamin D metabolites including epimers during pregnancy has been vastly questionable [42]. Recent studies are shedding light on a plausible biologically active role for epimers in vitamin D metabolism and hence its importance upon interpretation of serum 25(OH)D levels based on the measurement assay being used. Specific and accurate assays, namely, LC–MS/MS, separate epimers and thus provide a better diagnostic tool for the measurement of 25(OH)D during pregnancy [43].

## 5. Strengths and Limitations

To the best of our knowledge, this study is the first to evaluate the contribution of genetic variants of maternal and neonatal VDBP polymorphisms on adiponectin, irisin, and VDBP concentrations at birth, according to different cut-offs of vitamin D status, in maternal–neonatal dyads. Our findings, which were based on the inclusion of both maternal and neonatal polymorphisms in conjunction with the assessment of different 25(OH)D cut-offs, could pave the way for future investigations aiming to examine the potential role of variants of VDBP on maternal–neonatal VDBP and adipokine status. Results could be projected to guide future research for a personalized genotype-based approach that could be particularly valuable for metabolic profiles of future mothers and their offspring.

Despite the strengths of our study, there are a few limitations that should be acknowledged. First, the sample size was limited in its ability to identify additional differences in other maternal–neonatal cut-offs. Second, the causality between the examined correlates could not be confirmed by cross-sectional design. Third, all women were Caucasian, so the findings cannot be confidently projected to other populations. In addition, despite the implications of our results as a personalized genotype-based approach, one major limitation pertains to the practicality of applying such an approach in the current clinical setting in terms of cost-efficiency and feasibility. Hence, to monitor and secure adequate vitamin D status during pregnancy, vitamin D supplementation remains the norm in the clinical practice.

## 6. Conclusions

The findings in our study emphasize a potential role for VDBP genetic variants, CC genotype for rs2298850 and rs4588, in conjunction with a specific high cut-off of maternal 25(OH)D, in increasing maternal VBDP concentrations, hence providing a mechanistic rationale for aiming for specific cut-offs of vitamin D, after supplementation during pregnancy, in the daily clinical practice.

## Figures and Tables

**Table 1:** Demographics of mothers and neonates.

Mothers	
Number (*n*)	66
Age (years)	31.92 ± 6.08
Prepregnancy weight (kg)	67.56 ± 14.54
Term Weight (kg)	85.43 ± 14.30
Prepregnancy BMI (kg/m^2^)	24.91 ± 4.81
Term BMI (kg/m^2^)	29.62 ± 5.80
Duration of gestation (weeks)	38.80 ± 1.56
Small for gestational age (SGA,%)	0.04
Appropriate for gestational age (AGA,%)	0.96
Large for gestational age (LGA,%)	0.00
Daily dietary calcium intake during 3rd trimester (mg)	792.5 ± 334.0
Daily dietary vitamin D intake during 3rd trimester (mcg)	2.9 ± 1.2
**Neonates**	
Number (*n*)	66
Gender; females (*n* (%))	28 (0.42)
Height (cm)	50.48 ± 1.96
Weight (g)	3292.12 ± 414.25

**Table 2:** Neonatal serum biomarkers according to maternal vitamin D status.

		N	Neonatal VDBP (µg/mL)	N	Neonatal Adiponectin (µg/mL)	N	Neonatal Irisin (ng/mL)
Maternal vitamin D status	<25 nmol/L	17	470.29 ± 325.47 (426.69 ± 292.65)	16	20.56 ± 20.53 (20.95 ± 21.53)	10	141.03 ± 90.35
	>25 nmol/L	47	313.63 ± 176.35 (294.24 ± 125.52)	47	8.97 ± 13.24 (7.28 ± 7.73)	32	184.82 ± 186.36
	*p*-value; adjusted *p*; power		0.074 (0.085) * 41% ^ϕ^		0.048 (0.003) * 88% ^ϕ^		0.32
	<50 nmol/L	42	372.86 ± 271.59	41	12.00 ± 15.36	25	157.62 ± 139.61
	>50 nmol/L	22	321.61 ± 132.69	22	11.76 ± 17.67	17	199.06 ± 206.09
	*p*-value		0.41		0.96		0.48
	<75 nmol/L	53	365.10 ± 248.70	52	11.23 ± 13.99	33	182.44 ± 172.66
	>75 nmol/L	11	307.78 ± 136.31	11	15.19 ± 24.24	9	144.91 ± 158.43
	*p*-value		0.46		0.46		0.56

If the Levene’s test for equality of variances *p* > 0.05, then equal variances assumed (significant (2-tailed) *p*-values of *t*-tests are given). If the Levene’s test for equality of variances *p* < 0.05, then equal variances not assumed (significant (2-tailed) *p*-values of *t*-tests are given). * The data and *p*-values adjusted for maternal height (cm), BMI prepregnancy (kg/m^2^), BMI terminal (kg/m^2^), and weeks of gestation by one-way analysis of covariance (ANCOVA). ^ϕ^ Posthoc power analysis. Abbreviations: VDBP: Vitamin D-binding protein; 25(OH)D: 25-hydroxy-vitamin D. Digits in bold refer to significant effects.

**Table 3:** Neonatal serum biomarkers according to neonatal vitamin D status at birth and neonatal VDBP polymorphisms.

		Neonatal Vitamin D Status at Birth ≤ 25 nmol/L				Neonatal Vitamin D Status at Birth ≤ 50 nmol/L				Neonatal Vitamin D Status at Birth ≤ 75 nmol/L			
SNP	Neonatal Genotype	N	VDBP (µg/mL)	Adiponectin (µg/mL)	Irisin (ng/mL)	N	VDBP (µg/mL)	Adiponectin (µg/mL)	Irisin (ng/mL)	N	VDBP (µg/mL)	Adiponectin (µg/mL)	Irisin (ng/mL)
rs2298850	CC	14	400.07 ± 296.4	8.76 ± 10.6	117.44 ± 105.4	24	406.00 ± 293.6	8.76 ± 9.0	120.35 ± 115.2	29	402.94 ± 276.5	11.02 ± 13.3	126.96 ± 125.9 (152.40 ± 136.5)
	CG + GG	11	389.22 ± 297.3	18.57 ± 20.8	205.54 ± 198.5	26	324.81 ± 207.0	10.44 ± 15.2	202.16 ± 159.2	30	323.37 ± 193.0	10.93 ± 14.4	233.56 ± 191.3 (264.81 ± 214.6) *
	*p*-value adjusted *p*		0.93	0.19	0.27		0.26	0.64	0.11		0.20	0.98	0.04 0.091 *
rs4588	CC	13	406.69 ± 307.4	9.34 ± 10.8	117.44 ± 105.4	22	402.11 ± 306.5	9.97 ± 9.7	132.64 ± 125.6	27	399.54 ± 286.2	12.17 ± 13.7	136.18 ± 132.6
	CA + AA	12	382.95 ± 284.3	16.98 ± 20.4	117.44 ± 105.4	28	333.66 ± 202.4	9.33 ± 14.5	190.63 ± 157.3	32	331.21 ± 189.7	9.93 ± 13.8	223.31 ± 191.7
	*p*-value		0.84	0.28	0.27		0.35	0.86	0.27		0.28	0.54	0.11
rs7041	GG	7	407.68 ± 352.0	9.71 ± 14.1	127.62 ± 122.6	14	353.87 ± 260.3	9.19 ± 10.9	133.69 ± 130.5	15	341.49 ± 255.4	10.76 ± 12.1	130.2 ± 123.6
	GT + TT	18	390.47 ± 274.59	14.14 ± 17.0	165.44 ± 166.9	36	367.63 ± 253.8	9.79 ± 13.2	174.39 ± 149.7	44	369.64 ± 235.8	11.05 ± 14.4	194.3 ± 178.8
	*p*-value		0.89	0.55	0.68		0.86	0.88	0.48		0.70	0.95	0.30

If the Levene’s test for equality of variances *p* > 0.05, then equal variances assumed (significant (2-tailed) *p*-values of *t*-tests are given). If the Levene’s test for equality of variances *p* < 0.05, then equal variances not assumed (significant (2-tailed) *p*-values of *t*-tests are given). * The data and *p* values adjusted for maternal height (cm), BMI pre-pregnancy (kg/m^2^), BMI terminal (kg/m^2^), UVB exposure and weeks of gestation by One-way analysis of covariance (ANCOVA). Abbreviations: VDBP: Vitamin D binding protein; 25(OH)D: 25-hydroxy-vitamin D. Digits in bold refer to significant effects.

**Table 4:** Maternal serum biomarkers according to maternal vitamin D status and maternal VDBP polymorphisms.

		Maternal Vitamin D Status ≤ 25 nmol/L				Maternal Vitamin D Status ≤ 50 nmol/L				Maternal Vitamin D Status ≤ 75			
SNP	Maternal Genotype	N	VDBP (µg/mL)	Adiponectin (µg/mL)	Irisin (ng/mL)	N	VDBP (µg/mL)	Adiponectin (µg/mL)	Irisin (ng/mL)	N	VDBP (µg/mL)	Adiponectin (µg/mL)	Irisin (ng/mL)
rs2298850	CC	7	372.47 ± 79.49	4.74 ± 3.88	304.16 ± 495.99	17	384.16 ± 73.08	4.15 ± 2.92	353.43 ± 414.39	22	394.67 ± 71.29 (403.06 ± 4.72) *	4.81 ± 2.93	452.99 ± 513.74
	CG + GG	10	368.12 ± 6 1.91	5.02 ± 3.22	176.33 ± 134.89	25	365.70 ± 127.39	4.07 ± 2.84	277.50 ± 339.70	33	341.46 ± 121.86 (342.93 ± 64.36) *	3.82 ± 3.23	233.02 ± 298.82
	*p*-value adjusted *p* power		0.90	0.88	0.52		0.59	0.93	0.55		0.07 0.007 * 80% ^ϕ^	0.26	0.10
rs4588	CC	6	386.40 ± 77.15	5.13 ± 4.10	351.67 ± 525.59	16	390.11 ± 71.09	4.26 ± 2.98	377.31 ± 419.19	21	399.70 ± 68.92 (403.06 ± 64.72) *	4.92 ± 2.95	475.83 ± 517.28 (508.57 ± 559.87) *
	CA + AA	11	360.92 ± 63.40	4.76 ± 3.14	156.68 ± 136.70	26	362.74 ± 125.72	4.00 ± 2.80	265.75 ± 336.06	34	339.92 ± 120.33 (342.93 ± 64.36) *	3.77 ± 3.19	225.89 ± 296.20 (265.22 ± 332.14) *
	*p*-value adjusted *p* power		0.47	0.84	0.33		0.43	0.78	0.38		0.04 0.007 * 80% ^ϕ^	0.20	0.04 0.03 * 60% ^ϕ^
rs7041	GG	5	383.98 ± 86.01	5.61 ± 4.39	139.86 ± 94.03	11	379.45 ± 80.50	4.68 ± 3.50	215.67 ± 209.68 (198.10 ± 302.52) *	13	391.84 ± 82.84	4.89 ± 3.24	446.47 ± 561.90
	GT + TT	12	364.05 ± 61.42	4.58 ± 3.04	296.01 ± 437.13	31	370.94 ± 117.33	3.88 ± 2.58	340.29 ± 406.95 (178.58 ± 91.93) *	42	353.74 ± 112.89	4.02 ± 3.08	287.0 ± 356.91
	*p*-value adjusted *p*		0.60	0.59	0.45		0.72	0.10	0.06 0.96 *		0.27	0.38	0.26

If the Levene’s test for equality of variances *p* > 0.05, then equal variances assumed (significant (2-tailed) *p*-values of *t*-tests are given). If the Levene’s test for equality of variances *p* < 0.05, then equal variances not assumed (significant (2-tailed) *p*-values of *t*-tests are given). * The data and *p*-values adjusted for maternal height (cm), BMI prepregnancy (kg/m^2^), BMI terminal (kg/m^2^), UVB exposure, and weeks of gestation by one-way analysis of covariance (ANCOVA). ^ϕ^ Posthoc power analysis. Abbreviations: VDBP: vitamin D-binding protein; 25(OH)D: 25-hydroxy-vitamin D. Digits in bold refer to significant effects.

**Table 5:** Neonatal serum biomarkers according to maternal vitamin D status and maternal VDBP polymorphisms.

		Maternal Vitamin D Status ≤ 25 nmol/L				Maternal Vitamin D status ≤ 50 nmol/L				Maternal Vitamin D Status ≤ 75 nmol/L			
SNP	Maternal Genotype	N	Neonatal VDBP (µg/mL)	Neonatal Adiponectin (µg/mL)	Neonatal Irisin (ng/mL)	N	Neonatal VDBP (µg/mL)	Neonatal Adiponectin (µg/mL)	Neonatal Irisin (ng/mL)	N	Neonatal VDBP (µg/mL)	Neonatal Adiponectin (µg/mL)	Neonatal Irisin (ng/mL)
rs2298850	CC	8	550.44 ± 409.76	21.51 ± 20.13	104.42 ± 108.07	18	460.17 ± 349.52	12.55 ± 16.06	131.22 ± 169.93	23	437.54 ± 320.83	11.35 ± 14.59	139.29 ± 173.78
	CG + GG	9	399.04 ± 229.82	19.61 ± 22.28	177.64 ± 57.76	24	307.39 ± 175.37	11.57 ± 15.15	186.23 ± 96.46	30	309.56 ± 159.58	11.13 ± 13.76	223.04 ± 166.43
	*p*-value		0.38	0.86	0.22		0.10	0.84	0.34		0.09	0.96	0.17
rs4588	CC	7	584.21 ± 430.39	24.42 ± 19.84	104.42 ± 108.07	17	468.76 ± 358.31	13.22 ± 16.29	131.22 ± 169.93	22	443.15 ± 327.22	11.81 ± 14.76	139.29 ± 173.78 (508.57 ± 559.87) *
	CA + AA	10	390.54 ± 218.34	17.56 ± 21.73	177.64 ± 57.76	25	307.65 ± 171.68	11.13 ± 14.97	186.23 ± 96.46	31	309.70 ± 156.90	10.80 ± 13.64	223.04 ± 166.43 (265.22 ± 332.14) *
	*p*-value		0.30	0.53	0.22		0.10	0.67	0.34		0.09	0.80	0.17
rs7041	GG	5	735.48 ± 420.43	28.19 ± 22.62	55.57 ± 61.63 (67.60 ± 82.02) *	11	580.23 ± 404.75 (524.75 ± 331.56) *	17.00 ± 18.70	126.99 ± 212.45	13	572.62 ± 370.91 (526.26 ± 282.39) *	14.83 ± 17.89	150.78 ± 213.35
	GT + TT	12	359.79 ± 211.0	17.09 ± 19.63	177.66 ± 75.90 (177.66 ± 75.90) *	31	299.28 ± 157.60 (302.78 ± 177.35) *	10.17 ± 13.86	172.04 ± 93.80	40	297.65 ± 145.31 (297.16 ± 161.38) *	10.03 ± 12.48	196.20 ± 155.20
	*p*-value adjusted *p* power		0.12	0.33	0.04 ^♦^		0.04 0.05 * 50% ^ϕ^	0.21	0.46		0.02 0.01 * 75% ^ϕ^	0.29	0.50

If the Levene’s test for equality of variances *p* > 0.05, then equal variances assumed (significant (2-tailed) *p*-values of *t*-tests are given). If the Levene’s test for equality of variances *p* < 0.05, then equal variances not assumed (significant (2-tailed) *p*-values of *t*-tests are given). * The data and *p*-values adjusted for maternal height (cm), BMI prepregnancy (kg/m^2^), BMI terminal (kg/m^2^), UVB exposure, and weeks of gestation by one-way analysis of covariance (ANCOVA). **^♦^** Due to reduced number of cases in GG of rs7041, adjusted *p*-values could not be calculated. ^ϕ^ Posthoc power analysis. Abbreviations: VDBP: Vitamin D-binding protein; 25(OH)D: 25-hydroxy-vitamin D. Digits in bold refer to significant effects.

**Table 6:** Neonatal serum biomarkers according to maternal vitamin D status at birth and neonatal VDBP polymorphisms.

		Maternal Vitamin D Status at Birth < 25 nmol/L				Maternal Vitamin D Status at Birth < 50 nmol/L				Maternal Vitamin D Status at Birth < 75 nmol/L			
SNP	Neonatal Genotype	N	VDBP (µg/mL)	Adiponectin (µg/mL)	Irisin (ng/mL)	N	VDBP (µg/mL)	Adiponectin (µg/mL)	Irisin (ng/mL)	N	VDBP (µg/mL)	Adiponectin (µg/mL)	Irisin (ng/mL)
rs2298850	CC	11	438.79 ± 328.87	17.25 ± 17.82	143.24 ± 108.39	21	429.05 ± 304.32	11.24 ± 14.44	132.62 ± 115.01	27	406.74 ± 281.96	10.38 ± 13.04	132.92 ± 131.37 (164.56 ± 142.93)
	CG + GG	6	528.03 ± 341.32	27.85 ± 26.29	135.87 ± 37.76	21	316.68 ± 227.97	12.80 ± 16.62	189.45 ± 166.11	26	321.85 ± 205.29	12.14 ± 15.16	241.86 ± 200.61 (260.39 ± 239.40)
	*p*-value adjusted *p*		0.61	0.36	0.91		0.18	0.75	0.32		0.22	0.66	0.07 0.19 * 25% ^ϕ^
rs4588	CC	10	451.27 ± 343.90	18.86 ± 17.92	143.24 ± 108.39 (161.87 ± 105.76)	19	426.97 ± 320.20	12.90 ± 15.15	145.80 ± 124.76	25	403.38 ± 292.79	11.58 ± 13.62	143.17 ± 138.10
	CA + AA	7	497.46 ± 321.91	23.40 ± 25.92	135.87 ± 37.76 (135.87 ± 37.76)	23	328.16 ± 221.27	11.22 ± 15.86	172.67 ± 161.55	28	330.91 ± 200.87	10.90 ± 14.58	229.56 ± 201.54
	*p*-value		0.88	0.15	0.057 ^♦^		025	0.73	0.64		0.29	0.86	0.16
rs7041	GG	6	412.32 ± 387.53	14.51 ± 14.23	143.15 ± 106.03	10	376.99 ± 296.05	9.50 ± 12.49	159.27 ± 138.64	14	353.88 ± 260.30	9.19 ± 10.90	133.69 ± 130.52
	GT + TT	11	501.91 ± 302.14	24.19 ± 23.48	139.62 ± 89.11	32	371.58 ± 268.52	12.81 ± 16.28	156.98 ± 143.98	39	369.12 ± 247.78	11.98 ± 15.03	200.72 ± 185.13
	*p*-value		0.60	0.38	0.96		0.96	0.56	0.97		085	0.53	0.33

If the Levene’s test for equality of variances *p* > 0.05, then equal variances assumed (significant (2-tailed) *p*-values of *t*-tests are given). If the Levene’s test for equality of variances *p* < 0.05, then equal variances not assumed (significant (2-tailed) *p*-values of *t*-tests are given). * The data and *p*-values adjusted for maternal height (cm), BMI prepregnancy (kg/m^2^), BMI terminal (kg/m^2^), and weeks of gestation by one-way analysis of covariance (ANCOVA). **^♦^** Due to reduced number of cases in CA + AA of rs4588, adjusted *p*-values could not be calculated. ^ϕ^ Posthoc power analysis. Abbreviations: VDBP: vitamin D-binding protein; 25(OH)D: 25-hydroxy-vitamin D. Digits in bold refer to significant effects.

## Data Availability

The data presented in this study are available on request from the corresponding author.
